# Self-Power Dynamic Sensor Based on Triboelectrification for Tilt of Direction and Angle

**DOI:** 10.3390/s18072384

**Published:** 2018-07-22

**Authors:** Hyeonhee Roh, Inkyum Kim, Jinsoo Yu, Daewon Kim

**Affiliations:** Department of Electronic Engineering, Kyung Hee University, 1732 Deogyeong-daero, Giheung-gu, Yongin 17104, Korea; hyeonhee@khu.ac.kr (H.R.); inkyum.kim@khu.ac.kr (I.K.); yjinsoo23@khu.ac.kr (J.Y.)

**Keywords:** triboelectric sensors, self-powered systems, tilt sensors, orientation sensors

## Abstract

With the great development of the Internet of Things (IoT), the use of sensors have increased rapidly because of the importance in the connection between machines and people. A huge number of IoT sensors consume vast amounts of electrical power for stable operation and they are also used for a wide range of applications. Therefore, sensors need to operate independently, sustainably, and wirelessly to improve their capabilities. In this paper, we propose an orientation and the tilt triboelectric sensor (OT-TES) as a self-powered active sensor, which can simultaneously sense the tilting direction and angle by using the two classical principles of triboelectrification and electrostatic induction. The OT-TES device consists of a rectangular acrylic box containing polytetrafluoroethylene (PTFE) balls moved by gravity. The output voltage and current were 2 V and 20 nA, respectively, with a PTFE ball and Al electrode. The multi-channel system was adopted for measuring the degree and direction of tilt by integrating the results of measured electrical signals from the eight electrodes. This OT-TES can be attached on the equipment for drones or divers to measure their stability. As a result, this proposed device is expected to expand the field of TES, as a sensor for sky and the underwater.

## 1. Introduction

The great development of electronic technology brings the fourth industrial revolution that has compounded information and communication technology. “Internet of things”, which means electronics are connected with the Internet and can share all the information they have. The massive interchange and collection of information of machine to machine and machine to people interactions are conducted by different kinds of sensors.

With the use of sensors increasing exponentially, sensors have become independent and portable in the past few years. As the number of sensors increases, the extensive problem of batteries becomes serious. The trillions of batteries are restricted applications of sensors, causing inconvenience because they have a limited lifetime, replacing, and exchanging. To be suitable for future sensors, they must be operated with self-powered systems. In addition, the self-powered capability can extend the application in wireless sensors, such as health monitoring, robotic systems, patient monitoring, and national security [1].

In late 2012, the triboelectric nanogenerator (TENG) was invented as a new category of energy harvesting by Wang and his co-workers [2,3,4,5]. TENG produces electrical output based on the combination of two physical principles, electrostatic induction and contact electrification [1,6,7,8,9], which has not only high stability and high output performance, but is also simple, low-cost, robust, and an eco-friendly technology [10,11,12,13,14,15,16,17,18,19,20,21]. Since TENG respond to external mechanical conditions of impact [2,22], pressure, vibration [23,24], and human triggering with electrical signals [17,22,25,26,27,28,29,30], it can also operate as self-powered triboelectric sensors (TES). TES actively detects static and dynamic processes arising from mechanical stimulation by using the voltage and current output signals from triboelectrification [31]. There are many kinds of TES, such as a pressure sensor [32,33], biosensor, velocity sensor, motion sensor [34], vibration sensor, angle sensor, security sensor, and so on [35,36].

The tilt sensor has been widely used in various areas, such as architecture, cellular phones, airplanes, robots, and military applications [37]. Tilt sensors have been developed in a variety of approaches, for example, electrolytes [38], mercury [39], bubble [40], and thermal [41] approaches. However, electrolytes, such as KOH and mercury, are toxic, and bubble-level tilt sensors have a complex structure. Thermal tilt sensors are high power consumers. These existing commercial sensors commonly require an external power source and can be affected by temperature and humidity.

Here, in this work, we demonstrate a new type of self-powered triboelectric sensor that detects not only the tilting angle, but also the direction. The orientation and tilt triboelectric sensor (OT-TES), which consists of an acrylic container, polytetrafluoroethylene (PTFE) balls, and eight aluminum electrodes, detects the movement of a PTFE ball through electrical signals observed on eight Al electrodes. Due to the OT-TES being constructed with solid materials, it shows an insensitiveness to changes of temperature and humidity, maintaining a sensitive response to variation of the tilting angle. When the OT-TES device is tilted by 20 degrees with one PTFE ball, the output voltage and current are 2 V and 20 nA, respectively. Eight Al electrodes are located at the corner of each top and bottom surface of the device. Since each electrode detects the motion of PTFE balls, the multi-channel system is employed to sense the specific tilting state by combining the voltage changes of eight electrodes. OT-TES has the advantage of being insensitive to humidity and temperature as a tilt sensor, in addition to the simple and cost-effective fabrication method.

## 2. Materials and Methods

Fabrication of OT-TES: The acrylic box was made of acrylic, with a thickness of 2 mm. The size of the bottom surface was 15 cm × 19.5 cm and the height of the box was 3 cm. Four aluminum electrodes were located on the top and bottom outer surface at the corner, respectively. The total number of electrodes was 8. All electrode sizes were 5 cm × 5 cm and the thickness was 1 mm. The diameter of the PTFE balls was 6 mm. The balls occupied as much as 25% of the bottom surface.

Electrical measurement and multi-channel system: To set the tilting angle of the device, the protractor was used for setting the tilting angle (from 0° to 45°). When measuring only one electrode, the open-circuit voltage (*V_OC_*) and the short-circuit current (*I_SC_*) were measured by an electrometer (Keithley 6514). The Al electrode of the OT-TES was connected to the plus terminal of the Keithley 6514, and the minus terminal of the Keithley 6514 was connected to ground. To characterize the electrical output of the 8 electrodes and to protect the data acquisition board (DAQ) from high voltage generated from the OT-TES, a multi-channel system was designed. A multi-channel system consists of an electrometer (Keithley 6514), DAQ, resistor of 10 MΩ, and a capacitor of 47 nF. The DAQ received electrical signals reduced to a percentage from each electrode of the OT-TES after passing a resistor and a capacitor, which were connected in parallel with one terminal of the electrode. The eight Al electrodes of the OT-TES were connected to the plus terminal of the equipment and the last eight minus terminals of the multi-channel system were connected to one normal Al foil. In other words, all the minus terminals of the multi-channel system had the same ground. Since the speed of the tilt can affect the electrical output, each experiment was conducted under only two conditions, 0.5 Hz or 1 Hz frequency. In all experiments, the OT-TES was repeatedly tilted to generate a continuous signal. In this paper, the OT-TES was tilted (oscillated) in two directions, straight and diagonal directions. It can be confirmed by the image shown in the supplementary Figure S1.

## 3. Results and Discussion

### 3.1. OT-TES with One PTFE Ball

**Figure 1:**
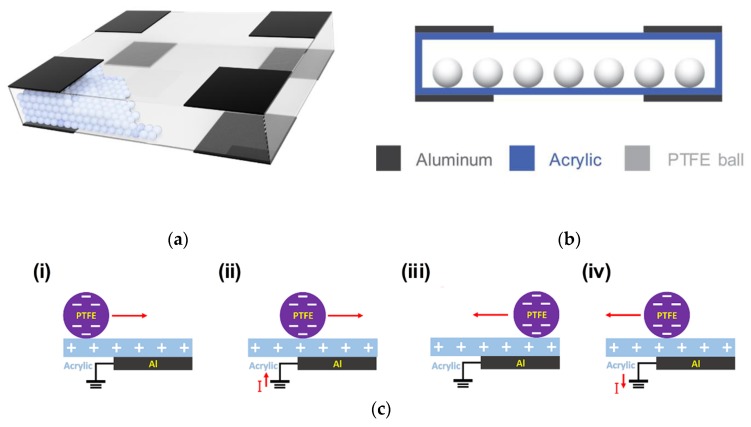
(**a**) Overall schematics of the orientation and tilt triboelectric sensor (OT-TES); (**b**) Cross-sectional view of the OT-TES; (**c**) Working mechanism of the OT-TES.

The overall schematic of the orientation and tilt triboelectric sensor (OT-TES) is shown in Figure 1a. The shape of the sensor is a rectangular parallelepiped. The OT-TES was composed of a transparent box (PMMA), white balls (PTFE), and eight electrodes (aluminum). The dimension of the transparent box was 15 cm × 19.5 cm × 3 cm and the size of each electrode was 5 cm × 5 cm. Four electrodes were located on each corner of bottom surface and the top surface, therefore the OT-TES had eight aluminum electrodes for detecting orientation and tilting angle. Figure 1b shows a cross-sectional view of the OT-TES. The diameter of the PTFE ball was 6 mm. The two materials, PTFE and acrylic, were chosen to induce electrification effectively. PTFE ball have a stronger ability to attract electrons than acrylic. As a result, the white balls (PTFE) tended to be negatively charged whereas the transparent box (PMMA) tended to be positively charged. The working mechanism of the OT-TES is schematically illustrated in Figure 1c. The fundamental operation principle modes of the OT-TES were based on the single-electrode mode [4,5,14]. When the device was tilted by an external force, the PTFE balls rolled along the device inner surface, which consisted of acrylic. As the negatively charged balls approached the Al electrode, the electrical potential of the Al electrode decreased. To reach an electrically neutral state, electrons flow from the Al electrode to the ground [20]. When the balls arrived at the end of the Al electrode, the surface charges reached the maximum value and the electrical potential became the minimum value. While the balls began to move away from the Al electrode, the opposite phenomenon occurred. As the PTFE balls, which have many electrons, drifts apart from the electrode, electrons flow into the aluminum electrode and the electrical potential increases.

**Figure 2:**
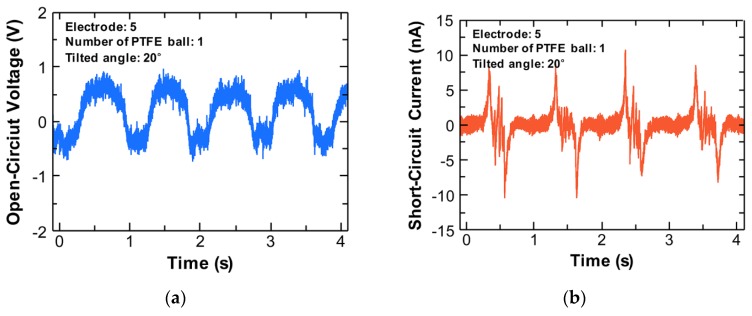
Electrical signals of a polytetrafluoroethylene (PTFE) ball from one Al electrode: (**a**) Open-circuit voltage; (**b**) Short-circuit current.

Figure 2a,b shows the open-circuit voltage (*V_OC_*) and the short-circuit current (*I_SC_*), respectively, of an OT-TES with one PTFE ball from one Al electrode. The device was tilted at 20 degrees and this experiment was conducted in a frequency of 1 Hz. Since the ball moves according to the tilting angle, the OT-TES has to remain in its original state to measure the changed angle. The electrical signal shows alternately positive and negative peaks centered at 0 V because both edges of the device are reciprocating up and down around the proposed reference axis. The state of 0 V means that the device has returned to its original state. As the ball approaches the electrode, the *V_OC_* rises, and the opposite result is detected when the ball moves away. The *V_OC_* and *I_SC_* were approximately 2 V and 10 nA, respectively.

### 3.2. Multi-Channel System

**Figure 3:**
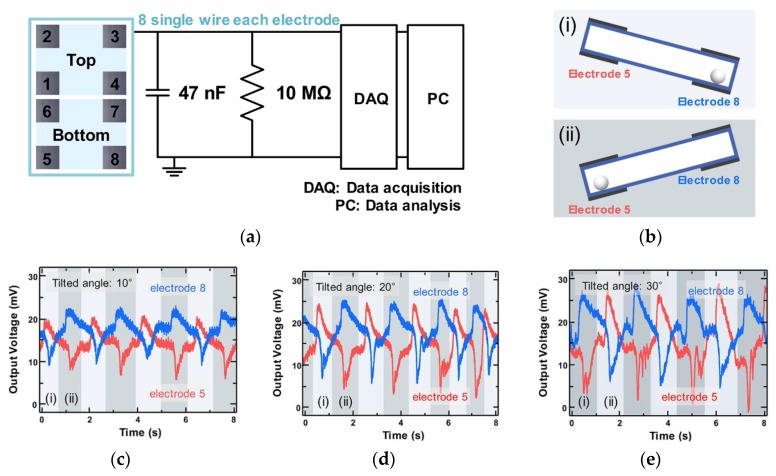
(**a**) Simple circuit diagram of a multi-channel system, including the eight electrodes located on the OT-TES. The data acquisition board (DAQ) conducts the data acquisition, and the personal computer (PC) analyzes the data; (**b**) Cross-sectional view of the OT-TES during tilting to the left and right side; (**c**–**e**) The DAQ output voltage measurements of a PTFE ball moving between two Al electrodes: (**c**) Tilt OT-TES at 10°; (**d**) tilt OT-TES at 20°; and (**e**) tilt OT-TES at 30°.

A multi-channel system was used to measure the electrical output of eight electrodes simultaneously. It is important to receive accurate information from the entire electrical signal of the OT-TES for advanced analysis. Figure 3a is a schematic of the multi-channel system circuit for signal processing. The 47 nF capacitor and 10 MΩ resistor convert and transmit the electrical signals to the data acquisition (DAQ) board with a small ratio at a constant rate. This process is essential because the DAQ can be damaged by high voltages that exceed 10 V. Finally, electrical signals were transmitted on personal computer (PC).

The following experiment was conducted to investigate the influence of the external force on the OT-TES. Depending on the external environment or force, PTFE balls in the OT-TES roll along the surface and make electrical output at the corresponding electrode. Two conditions were prepared, which are shown in Figure 3b. The DAQ output voltage of electrode 5 and 8 with a PTFE ball and measured by the multi-channel system are shown in Figure 3c–e. In this experiment, the tilting range was from 10 to 30 degrees and a multi-channel system was used.

Figure 3b shows a cross-sectional view of the OT-TES during tilting to the left and right side. When a PTFE ball rolls from electrode 5 to 8, the PTFE ball gets closer to electrode 8 and further away from electrode 5. As shown in Figure 3c–e, the PTFE ball produces an opposite waveform for each electrode perfectly (electrode 5: Red, electrode 8: Blue). Since the PTFE ball has a uniformly accelerated motion in a straight line along the long side of the bottom layer of the PMMA box, the moving distance is 19.5 cm. At 10, 20, and 30 degrees, the time required for the ball to move is 1, 0.9, and 0.7 s, respectively. As a result, the speed was increased to 19.5, 21, and 27 cm/s, and the accelerations were increased to 19.5, 23.4, and 38.6 cm/s^2^. The large tilting angle causes the PTFE ball to move faster and induces a greater change in the output voltage. Comparing Figure 3c–e, the DAQ output voltage was increased by about 8 mV from 20 to 28 mV. Since a reciprocating motion must be generated within a constant time, the tilting speed changes according to the tilting angle. As a result, only the variation of angle affects the tilting speed. For this reason, a large change in the current related to the ball speed affects the output voltage according to Ohm’s law in a multi-channel system.

### 3.3. OT-TES with Multiple PTFE Balls

Multiple balls were used to subdivide the electrostatic induction on the top electrode and to obtain an accurate tilting angle. The OT-TES had eight electrodes located on the four corners of the top and bottom surfaces. As the tilting angle increases, PTFE balls roll from one side to the opposite side and accumulate multiple layers at the corner. The multiple layers of balls created at a larger angle have a greater effect on the top electrode than a single layer of balls. PTFE balls could be stacked up to five layers because the height of the PMMA box was 30 mm and the diameter of a PTFE ball was 6 mm.

Also, the number of PTFE balls affects the output voltage located on the bottom surface. As the number of PTFE balls increased, electrification was more activated due to the increased contact surface between the acrylic plate and PTFE balls. To optimize the area occupied by the PTFE balls on the bottom plate, the output voltage was observed by varying the number of balls. The device was tiled 20° and the 0.5 Hz condition was applied.

**Figure 4:**
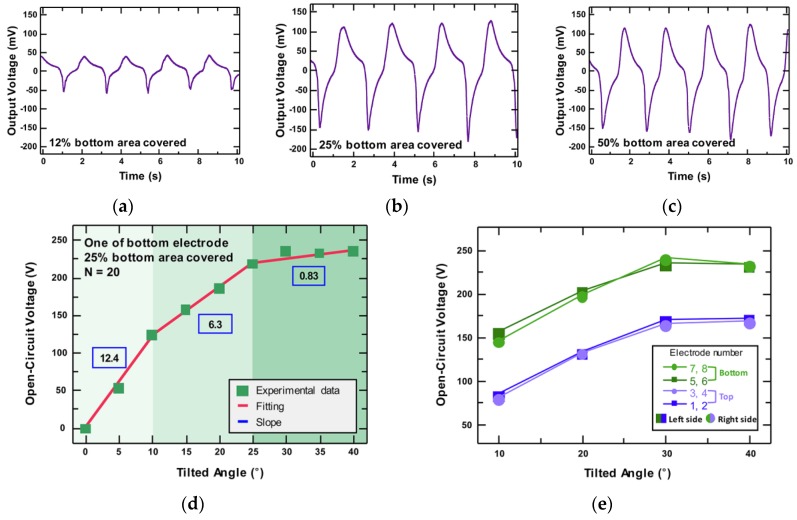
(**a**–**c**) The DAQ output voltage according to the number of PTFE balls occupying the bottom surface. The tilting angle and frequency are 20° and 0.5 Hz, respectively: (**a**) The amount of balls filled 12% of the bottom surface; (**b**) The amount of balls filled 25% of the bottom surface; (**c**) The amount of balls filled 50% of the bottom surface; (**d**) The open-circuit voltage of the bottom side with 25% amount of balls according to the tilting angle; (**e**) The open-circuit voltage of the bottom side and the top side.

12%, 25%, and 50% of the bottom surface area was covered with a single layer of PTFE balls in the order of Figure 4a–c, respectively. The DAQ output voltage was 0.1 V at 12%, 3 V at 25% (Figure 4b), and saturated from 25%. Even if the number of balls was increased to 50%, the maximum output voltage value did not change. The length of the bottom surface was 15 cm × 19.5 cm, so the dimension of the bottom surface was 292.5 cm^2^. The length of the square shaped aluminum electrode was 5 cm, so this electrode occupied a quarter of the length of the bottom surface. When the PTFE balls existed at the bottom area over 25%, the Al electrode was completely covered and this proportion was the most optimized amount of PTFE balls. The amplitude of the output voltage was no longer affected by increasing the number of PTFE balls.

To determine the voltage changes relative to the angle, the OT-TES was tilted from 0° to 40° and 25% of the bottom area was covered by PTFE balls. Figure 4d shows the *V_OC_* of one bottom electrode. When the *V_OC_* rose to 10°, with an increase rate of 12.4 V/degree, the increase rate decreased to 6.3 V/degree with 25°. Finally, beyond 25°, the growth rate was sharply decreased to 0.83 V/degree and saturated. This means that the *V_OC_* had reached a measurable peak. The larger size of the OT-TES could measure larger ranges than this proposed device.

The eight electrodes could be paired in various ways, with respect to the movements of the OT-TES. For example, the electrodes can be divided into two different groups as the left side and right side or top surface and bottom surface. Due to the ability to detect all tilting directions, the positions of the OT-TES device can be obtained by analyzing the amplitude and waveform of the electrical signals. In the case of Figure 4e, the *V_OC_* of the bottom electrodes were larger than the top surface electrodes. The electrodes were paired with the upper and lower parts (upper electrodes: 1, 2, 3, and 4; lower electrodes: 5, 6, 7, and 8). Because of gravity, the PTFE balls were always in contact with the bottom surface, which was located at the lower side. As a result, more triboelectric charges were induced at the lower side than the upper side, producing a high voltage. If the electrodes were divided with close voltage levels into two groups, the top surface would be represented as a high voltage and the bottom surface would be represented as a low voltage.

### 3.4. Comprehensive Analysis of OT-TES

To verify the applicability of the OT-TES, an experiment was carried out to investigate the direction and tilting angle by analyzing the signal of eight electrodes in a complex situation. The experiment was constructed in two ways by tilting the OT-TES in straight and diagonal directions. Supplementary Figure S1 shows the tilting directions.

**Figure 5:**
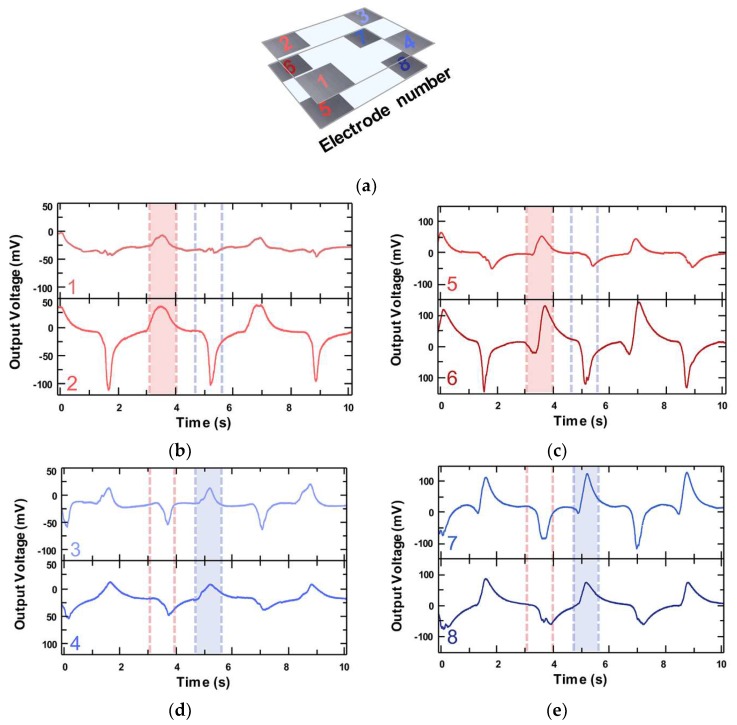
(**a**) The schematic of the OT-TES and each electrode number. (**b**–**e**) Voltage measurements of the OT-TES from eight Al electrodes when the tilt is 45 degrees to the left and right side and around to the center of the OT-TES: (**b**) The DAQ output voltage of electrode 1 and 2, and (**c**) the DAQ output voltage of electrode 5 and 6. (**d**) The DAQ output voltage of electrode 3 and 4, and (**e**) the DAQ output voltage of electrode 7 and 8.

At first, the OT-TES was tilted from 10° to 45° in a straight direction, and the PTFE balls reached several electrodes simultaneously. The location of electrodes can be checked through Figure 5a. While the right side was down, the PTFE balls were gathered at the right side by gravity. Eventually, the output voltage was increased at the right side electrode (3, 4, 7, and 8) while the voltage at the left side electrode (1, 2, 5, and 6) was decreased. Moreover, when the OT-TES device was tilted, the electrodes located underneath showed an increasing voltage, and the upper side electrodes showed the opposite result. The output voltage signals of the eight channels according to the tilting direction is shown in Figure 5b–e. The average output voltage of the bottom electrode (5 to 8) was 200 mV and the average output voltage of the top electrode (1 to 4) was 100 mV. The output voltages of the bottom side were doubled compared to that of the top surface. As mentioned above, the OT-TES can operate in any direction and utilize any side of electrode pairs. By applying this point, we can approximate the tilting direction and angle from detecting the signal generating electrodes. The result of the output voltage difference according to the angle can be seen in supplementary Figure S2a–d. Supplementary Figure S2a,b and S2c,d show the output voltage when tilting 10° and 20°, respectively.

In Figure 5b–e, the channel can be divided into two groups according to the shape of the output voltage. Waveforms of channel 1, 2, 5, and 6 (red color), and 3, 4, 7, and 8 (blue color), respectively, show the similar shape of the output voltage. The output voltages of each pair were elevated simultaneously. The electrodes corresponding to the two pairs of channels were located at each end of the device. When one side goes down, naturally, the other side goes up.

As shown in supplementary Figure S3, if the OT-TES was tilted diagonally and when the electrode 2 was down, the PTFE balls collected at electrodes 2 and 6. Eventually, the output voltage was increased at electrodes 1, 2 5, and 6 (red color), while the voltage at electrodes 3, 4, 7, and 8 (blue color) was decreased. Since the OT-TES is tilted only in a diagonal direction which is electrodes 2,4,6, and 8 are located, electrodes 1,3,5, and 7 are less affected by PTFE balls. As a result, electrodes 1,3,5, and 7 have a very small output voltage compared to electrodes 2,4,6, and 8. The output voltage for the straight tilt (10° and 20°) is illustrated in the supplementary Figure S4. In this figure, the tilting direction and degrees according to the voltage waveform and voltage amplitude, respectively, were confirmed.

## 4. Conclusions

In summary, we proposed a self-powered tilt sensor based on triboelectric effect and electrostatic induction. Since the OT-TES is consisted of solid materials, such as a PMMA box and PTFE balls, the OT-TES has the advantages of being simple, low-cost, and robust. Eight electrodes, which are located at the corners of the top and bottom outer surface of the acrylic box, were used for classifying the tilting direction and angle of the device. A multi-channel system can represent the electrical signals from each of the eight electrodes, respectively, for any users to enable an improved analysis. The amplitude and waveform of the output voltage represented the tilting angle and direction of the device. This study further expands the application of self-powered sensors by proposing a tilt sensor that is harmless to the human body and shows greater independence from time, humidity, and temperature than generally existing commercial tilt sensors.

## Supplementary Materials

The following are available online at http://www.mdpi.com/1424-8220/18/7/2384/s1, Figure S1: The tilting direction of the OT-TES, Figure S2: The DAQ output voltage of the OT-TES when tilting the device in the straight direction, Figure S3: The DAQ output voltage of the OT-TES from eight Al electrodes when tilting the device in the diagonal direction, Figure S4: The DAQ output voltage of the OT-TES when tilting the device in the diagonal direction.
