# Supplementary material for: Self-Power Dynamic Sensor Based on Triboelectrification for Tilt of Direction and Angle
Source: Sensors (Basel). 2018 Jul 22;18(7):2384. doi: 10.3390/s18072384 (PMC6068663; doi:10.3390/s18072384)
Supplement: Supplementary file 1 [file sensors-18-02384-s001.zip › sensors-325703-SI.pdf]

Supporting Information

Self-Power Dynamic Sensor Based on Triboelectrification for Tilt of Direction and Angle

Hyeonhee Roh, Inkyum Kim, Jinsoo Yu and Daewon Kim *

Department of Electronic Engineering, Kyung Hee University, 1732 Deogyeong-daero, Giheung-gu, Yongin 17104, Korea; hyeonhee@khu.ac.kr (H.R.); inkyum.kim@khu.ac.kr (I.K.); yjinsoo23@khu.ac.kr (J.Y.)

* Correspondence: daewon@khu.ac.kr; Tel.: +82-31-201-2567

Table of Contents

1. **The tilting direction of the OT-TES**
2. **The DAQ output voltage of the OT-TES when tilting the device in the straight direction**
3. **The DAQ output voltage of the OT-TES from eight Al electrodes when tilting the device in the diagonal direction**
4. **The DAQ output voltage of the OT-TES when tilting the device in the diagonal direction**

1. The tilting direction of the OT-TES

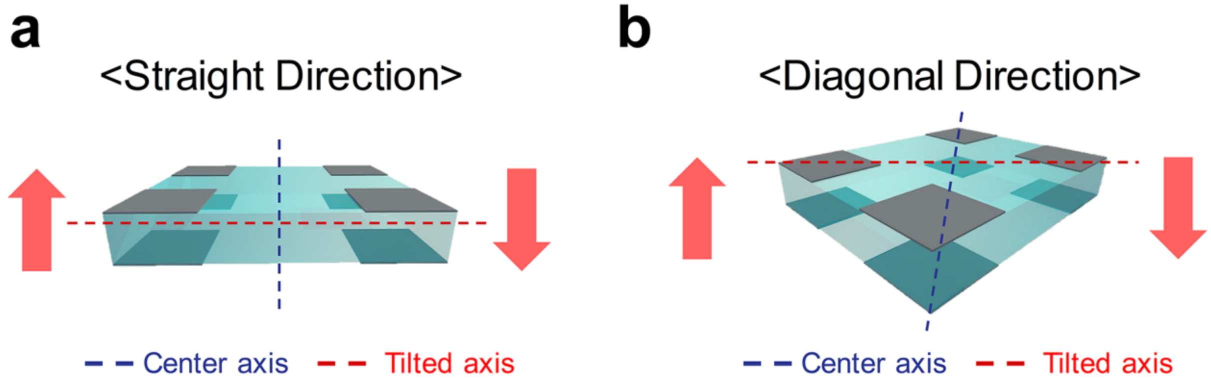

Figure A1. Two ways of the tilting direction (**a**) Straight direction (**b**) Diagonal direction.

2. The DAQ output voltage of the OT-TES when tilting the device in the straight direction

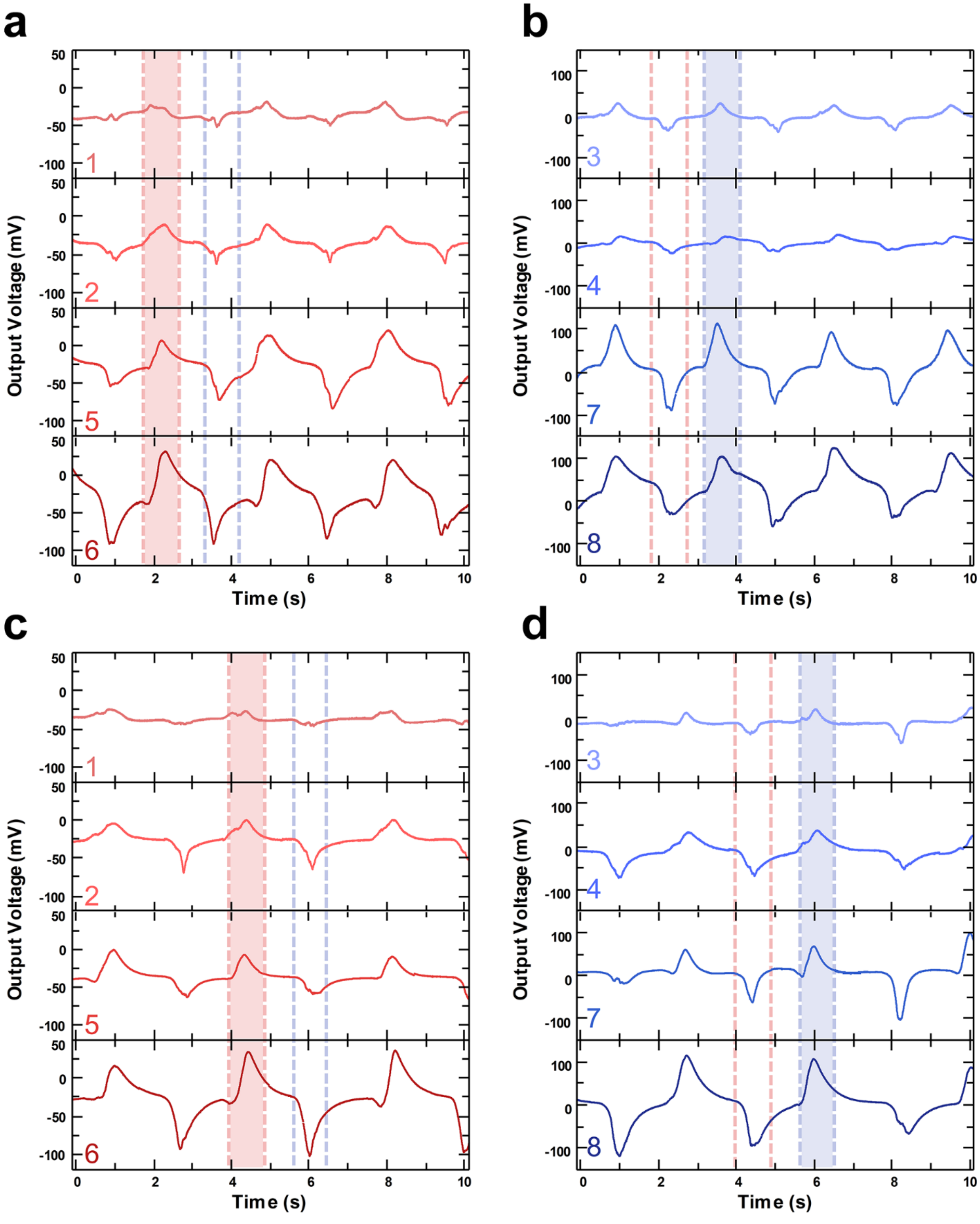

Figure A2. Voltage measurements of the OT-TES from eight different Al electrodes that

have adjusted tilt angle in the straight direction. (**a**) The DAQ output voltage of the left side electrodes at 10° (**b**) The DAQ output voltage of the right side electrodes at 10° (**c**) The DAQ output voltage of the left side electrodes at 20° (**d**) The DAQ output voltage of the right side electrodes at 20°.

3. The DAQ output voltage of the OT-TES from eight Al electrodes when tilting the device in the diagonal direction

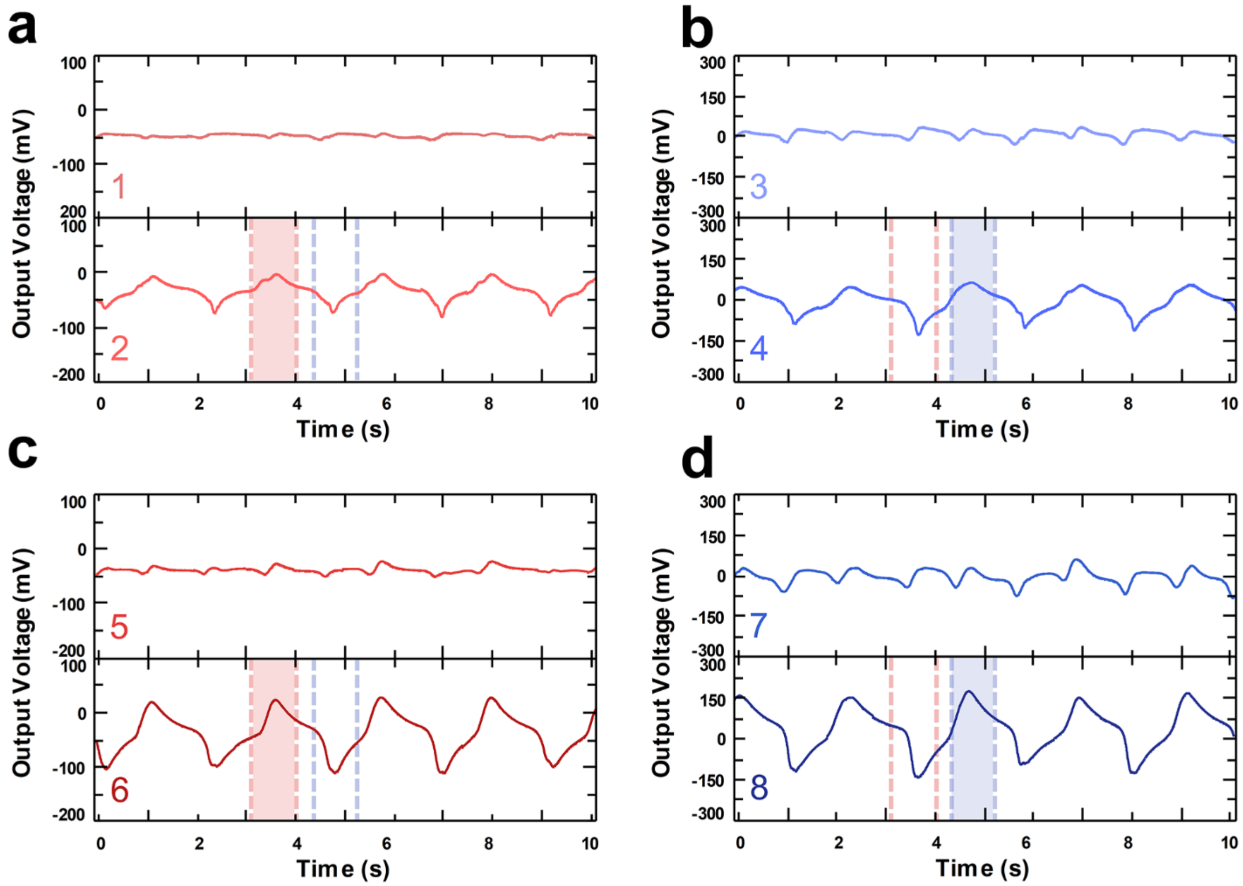

Figure A3. Voltage measurements of the OT-TES from eight Al electrodes that have 45 tilting degree in the diagonal direction: (**a**) The DAQ output voltage of the electrode 1 and 2 (**b**) The DAQ output voltage of the electrode 3 and 4 (**c**) The DAQ output voltage of the electrode 5 and 6 (**d**) The DAQ output voltage of the electrode 7 and 8.

4. The DAQ output voltage of the OT-TES when tilting the device in the diagonal direction

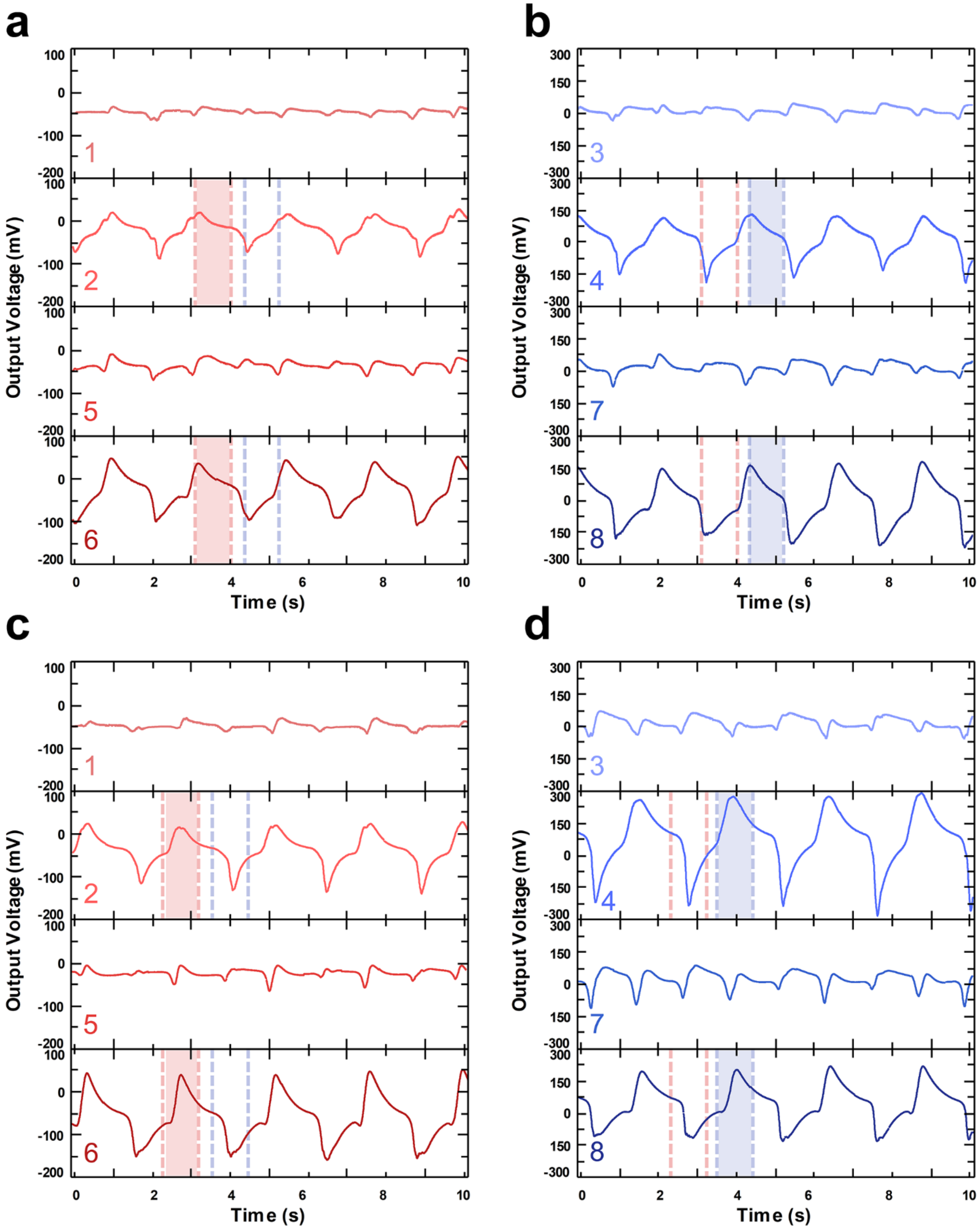

Figure A4. Voltage measurements of the OT-TES from eight different Al electrodes that have adjusted tilt angle in diagonal side. (**a**) The DAQ output voltage of the left side electrodes at 10° (**b**) The DAQ output voltage of the right side electrodes at 10° (**c**) The DAQ output voltage of the left side electrodes at 20° (**d**) The DAQ output voltage of the right side electrodes at 20°.
